# Does land cover affect the growth rate of COVID-19? Rethinking sustainable habitat from the One Health perspective using data from 12 cities during lockdown in Hubei Province, China

**DOI:** 10.3389/fpubh.2025.1682820

**Published:** 2026-01-14

**Authors:** Yidan Huang, Xiaomei Yuan, Jingwei Zhao

**Affiliations:** 1 State Key Laboratory of Subtropical Building and Urban Science, South China University of Technology, Guangzhou, China; 2 School of Architecture, South China University of Technology, Guangzhou, China; 3 School of Architecture and Design, China University of Mining and Technology, Xuzhou, China

**Keywords:** COVID-19, land cover, sustainable habitat, growth rate of infectious diseases, One Health (OH)

## Abstract

**Background:**

The COVID-19 pandemic has drawn attention to the interconnected roles of environmental conditions and public health beyond conventional medical explanations. The One Health (OH) perspective offers a collaborative and interdisciplinary perspective that integrates humans, animals, plants, and their shared environment to achieve optimal health outcomes. The 12 cities in Hubei Province that experienced lockdown during the peak phase of COVID-19 (February 1 to March 4, 2020) provided unique samples. In this study, land cover was selected as the environmental variable, and the COVID-19 growth rate was used as the infectious disease indicator to examine their relationship, thereby investigating the potential role of environmental factors in epidemic control.

**Methods:**

The Least Absolute Shrinkage and Selection Operator (LASSO) regression was used to identify the most influential variables for subsequent analyses. Spatial autocorrelation was assessed using Moran’s I in RStudio, while spatial dependence was explicitly modeled through the Spatial Autoregressive (SAR) and Spatial Lag of X (SLX) models to evaluate the effects of explanatory variables while accounting for spatial interactions. All results were interpreted within the One Health perspective, considering the source of infection, routes of transmission, and susceptible populations.

**Results:**

LASSO regression identified wetland, cultivated land, orchard land, forest land, and population density as the main factors associated with the COVID-19 growth rate. Wetland coverage exhibited a significant positive association with growth rate, whereas cultivated land showed a negative but marginally significant relationship. Orchard land and forest land were associated with weak negative effects.

**Conclusion:**

The statistical results indicate that variations in land cover influence the growth rate of COVID-19 cases, suggesting that environmental management, including wetland and wastewater control, agricultural landscape configuration, forest vegetation preservation, and control population density, may help mitigate infectious disease growth. From the One Health perspective, sustainable habitat design and planning strategies and land use policies were proposed for future research.

## 1 Introduction

In the post pandemic era, there is a growing awareness that the COVID-19 pandemic was not limited to the medical field, and we must situate it within wider environmental and public health issues. One Health (OH) is recognized as a collaborative, multisectoral, interdisciplinary perspective to connect people, animals, plants, and their common environment to achieve optimal health and well-being outcomes (1). However, currently published research utilizing the OH perspective has tended to focus primarily on animal or zoonotic diseases (2), and using environmental integration as a health tools into the OH perspective lags behind the integration of clinical data on human and animal health (3). Additionally, a large portion of the current research on COVID-19 continues to focus on medical issues such as controlling and treating infectious diseases (4). However, the emergence and mutation of the SARS-CoV-2 virus highlights its limited ability to overcome the threat of deadly and frequent outbreaks of infectious diseases (5). Prevention is well known as a better strategy than treatment, and there is a pressing need to rethink environmental sustainability and resilience from the OH perspective.

Human-to-human transmission has been identified as a key mechanism in the spread of COVID-19. This understanding led to a widespread perception that densely populated and highly connected environments facilitate viral transmission, a notion supported by several studies (6–8). For example, the poorest 60% of India’s urban population live in only about 72 square feet of floor space per person, a condition that has contributed to large-scale viral spread in the country (9). Nevertheless, in contrast to the majority of existing research, a study have indicated that population density is not statistically associated with COVID-19 transmission (10). These mixed findings suggest that population density alone cannot fully explain the dynamics of COVID-19 transmission. Instead, it likely interacts with socioeconomic, environmental, and institutional factors, underscoring the need for further investigation into the complex relationship between population density and infectious disease spread.

The severity of COVID-19 infection is closely associated with demographic characteristics such as age and preexisting comorbidities, including asthma, cardiovascular disease, and diabetes (11, 12). A number of studies have confirmed that individuals with weakened immune systems are more susceptible to COVID-19 infection (13, 14). In most infectious diseases, clinical variability largely depends on the host immune response to the pathogen (15). In addition, recent evidence indicates that individuals with lower levels of physical activity tend to experience more severe symptoms following COVID-19 infection (16). These findings suggest that biological and behavioral characteristics play a critical role in shaping individual vulnerability to the disease.

Beyond biological and demographic determinants, numerous studies have highlighted the influence of socioeconomic and ethnoracial disparities on COVID-19 infection and mortality rates (17–19). Early reports from the United States revealed that COVID-19 outcomes were strongly associated with long-standing social determinants of health, including race/ethnicity and socioeconomic inequality (20). A wide of factors have been proposed, such as social risks including low socioeconomic status, crowded housing, and dependence on public transportation (21). These findings underscore that COVID-19 is not only a biomedical phenomenon but also a manifestation of deeply entrenched structural inequities within societies.

During the COVID-19 pandemic, environmental variables were found to be critical. Infectious viruses, such as coronaviruses, are known to survive and persist for extended periods in suitable environments outside their hosts (22). While human-to-human contact remains a dominant route, growing evidence suggests that airborne transmission plays a substantial and sustained role in the spread of SARS-CoV-2 (23). Studies have indicated that higher concentrations of air pollutants and certain climatic conditions can prolong the survival time of SARS-CoV-2 in the atmosphere, thereby increasing the likelihood of infection (24). Air pollution has also been associated with increased COVID-19–related mortality and may contribute to higher transmission rates (25). Empirical evidence from multiple regions demonstrates that particulate matter, particularly PM2.5 and PM10, is significantly correlated with both infection and hospitalization rates for respiratory diseases (26).

The role of land cover in driving infectious diseases is well-known and is a matter of concern (27), because of its association with broader environmental factors (28). The fundamental definition of land cover is the observed physical cover present on the Earth’s surface (29). Changes in land cover provide the most direct reflection of the interaction between human activities and ecosystems (30). Since the COVID-19 pandemic, studies related to COVID-19 and the environment have garnered increasing attention. Some scholars have evaluated the links between urban development and COVID-19 growth rates, including factors such as built-up land areas (31), subways, wastewater and residential waste (32), built environment density, open space (33), and city size (34). Owing to the single dimension of the environment, the actual achievement is still limited. Furthermore, published research has resulted in a variety of topic-specific studies on land cover, including its relationship with mental health (35), the impact of meteorological factors on infection risk (36), virus transmission in the urban water supply cycle (37), built environment factors and infection rates (38), urban transport and infection rates (39), nature-based solutions (40), COVID-19 morbidity in rural and urban areas (41), and the construction of outbreak-preventive cities (42). While some scholars have also considered the impact of the environment on COVID-19, few have examined land cover as an integrated environmental system rather than as isolated components. For example, scholars have extensively discussed the impacts of built environment (33), build-up areas (43), and high-density districts (44). Nevertheless, these attempts to establish a link between the dissemination of COVID-19 and land cover were incomplete during its spread. Moore et al. (100) suggested that cases of COVID-19 may also be associated with broader landscape features. OH provides a valuable perspective for analyzing the complex relationships of the whole and offers possibilities for rethinking urban planning and design techniques (45) to improve sustainable habitats. A literature review thus indicates that it is meaningful to examine the relationship between land cover and COVID-19. However, a major challenge remains the lack of comparable peak-period COVID-19 datasets necessary for conducting such analyses.

**Figure 1:**
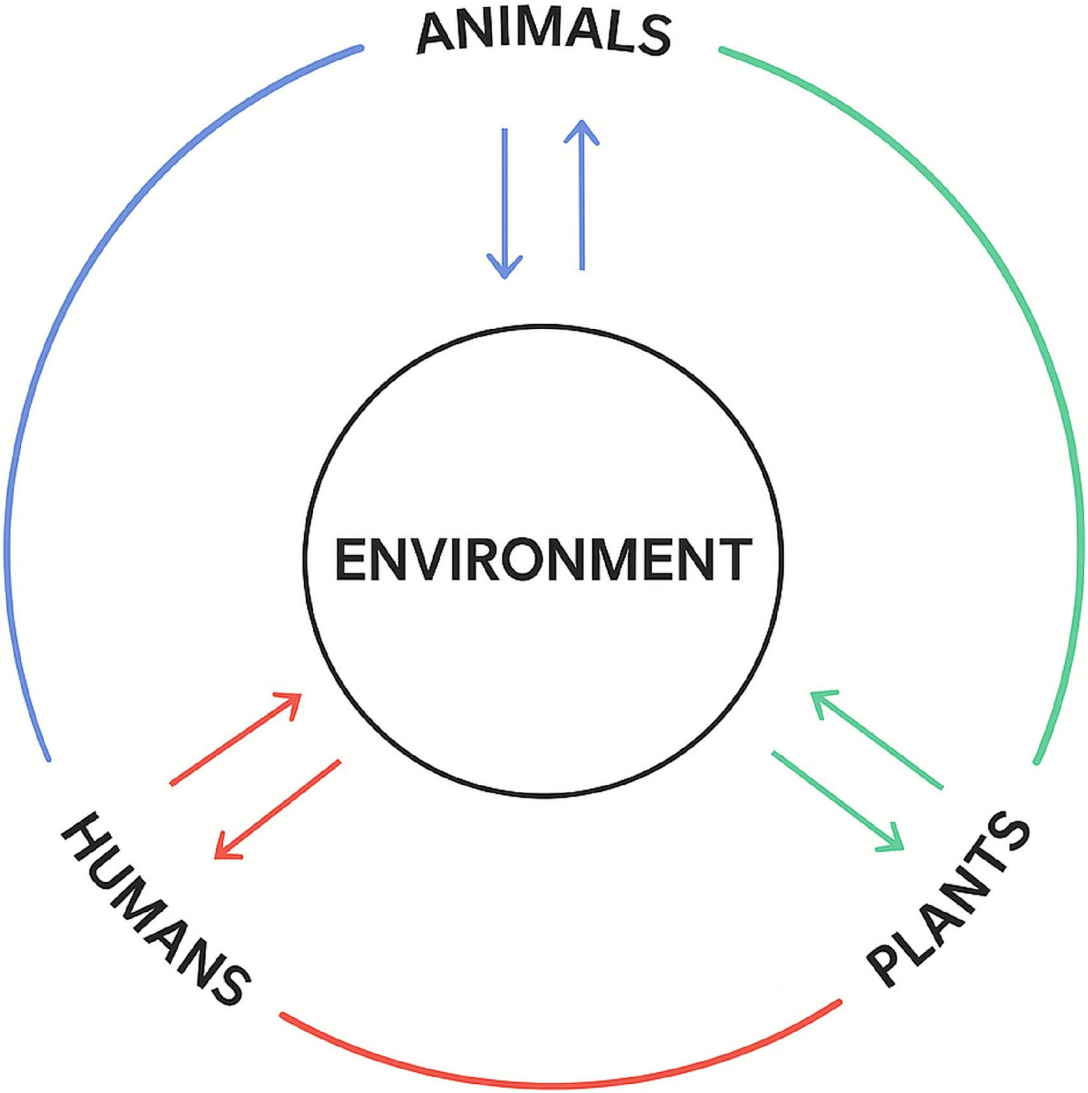
Human-animal–plant-environment interface from the One Health perspective.

As shown in Figure 1, the environment serves as a fundamental medium that accommodates and mediates the interactions among humans, animals, and plants within the OH perspective (46, 47). Since its inception, the One Health (OH) approach has emphasized collaboration among the Food and Agriculture Organization of the United Nations (FAO), the World Health Organization (WHO), and the World Organisation for Animal Health (WOAH, formerly OIE) to address risks at the human–animal–plants interface (47). In February 2021, these organizations invited the United Nations Environment Programme (UNEP) to join, reaffirming the importance of integrated and coordinated environmental action within the OH framework. As noted by Inger Andersen, Executive Director of the United Nations Environment Programme (UNEP), the establishment of the Quadripartite Alliance in 2022 marked a global shift from reactive disease control toward proactive, cross-sectoral prevention (48). The scope of One Health has since expanded beyond zoonoses to encompass the broader environmental determinants of health. Although the world has gradually moved beyond the acute phase of the COVID-19 pandemic, ongoing threats such as monkeypox, Ebola, antimicrobial resistance, ecological degradation, and climate change continue to underscore the enduring global relevance of this approach.

While global economic growth has significantly improved human well-being, it has often come at the cost of ecosystem integrity and environmental health. The above points clearly rethink the fact that COVID-19 is not an isolated phenomenon. Land-use expansion, rapidly changing farming systems, ecosystem alteration, forest encroachment, and high-density living environments are the key factors that increase the common risk of human–animal–plants interfaces (46). These risks are further exacerbated by rapid urbanization, inadequate waste management, and the intensifying climate crisis. Environmental degradation and the erosion of ecosystem services disrupt the balance among human, animal, and natural systems, altering pathogen transmission dynamics and heightening the likelihood of cross-species spillover (49). Pollutants generated by human activities, such as urbanization and industrialization, compromise the immune systems of both humans and animals (50), thereby amplifying the impact of pathogens (51). Researchers have hypothesized that the environment played an early and critical role in the transmission of COVID-19 (23). Therefore, as illustrated in Figure 1, this study adopts the One Health perspective as its theoretical foundation, emphasizing that human, animal, and plants health are interdependent components of the same shared environment, collectively shaping a sustainable and resilient future.

In summary, this study hypothesizes that land cover and other potential influencing factors—such as population density, socioeconomic indicators, healthcare capacity, and air pollution—exert significant effects on the growth rate of COVID-19 cases. Using data from 12 cities during the lockdown period, this study systematically integrates evidence from the fields of public health and environmental science, reassessing environmental design, policy, and planning through the lens of the One Health perspective. The primary objective is to elucidate how land cover, as a tangible environmental medium supporting human, animal, and plant interactions, shapes the dynamics of COVID-19, thereby informing strategies for the development of more resilient and sustainable habitats.

## 2 Materials and methods

### 2.1 Sample sites

Hubei Province, located in central China (29°01′53″–33°6′47″N, 108°21′42″–116°07′50″E), lies along the middle reaches of the Yangtze River and serves as an important inland transportation hub. The province covers an area of approximately 185,900 km^2^ and exhibits diverse topography, including extensive plains as well as abundant forested and wetland areas, resulting in a complex land cover composition. Hubei experiences a humid subtropical monsoon climate with four distinct seasons, an average annual temperature of 15–17 °C, and annual precipitation ranging from 800 to 1,600 mm. Economically, Hubei is a leading province in central China, with a diversified industrial structure and substantial agricultural output, particularly in rice and freshwater aquaculture. In summary, the 12 cities in Hubei were selected as study sites primarily because of the coexistence of plains and mountain-forest ecosystems, dense water networks, humid climate, diverse economic structure, and rich ecological resources, making the province a representative region for investigating the environmental determinants of COVID-19 growth rates.

**Figure 2:**
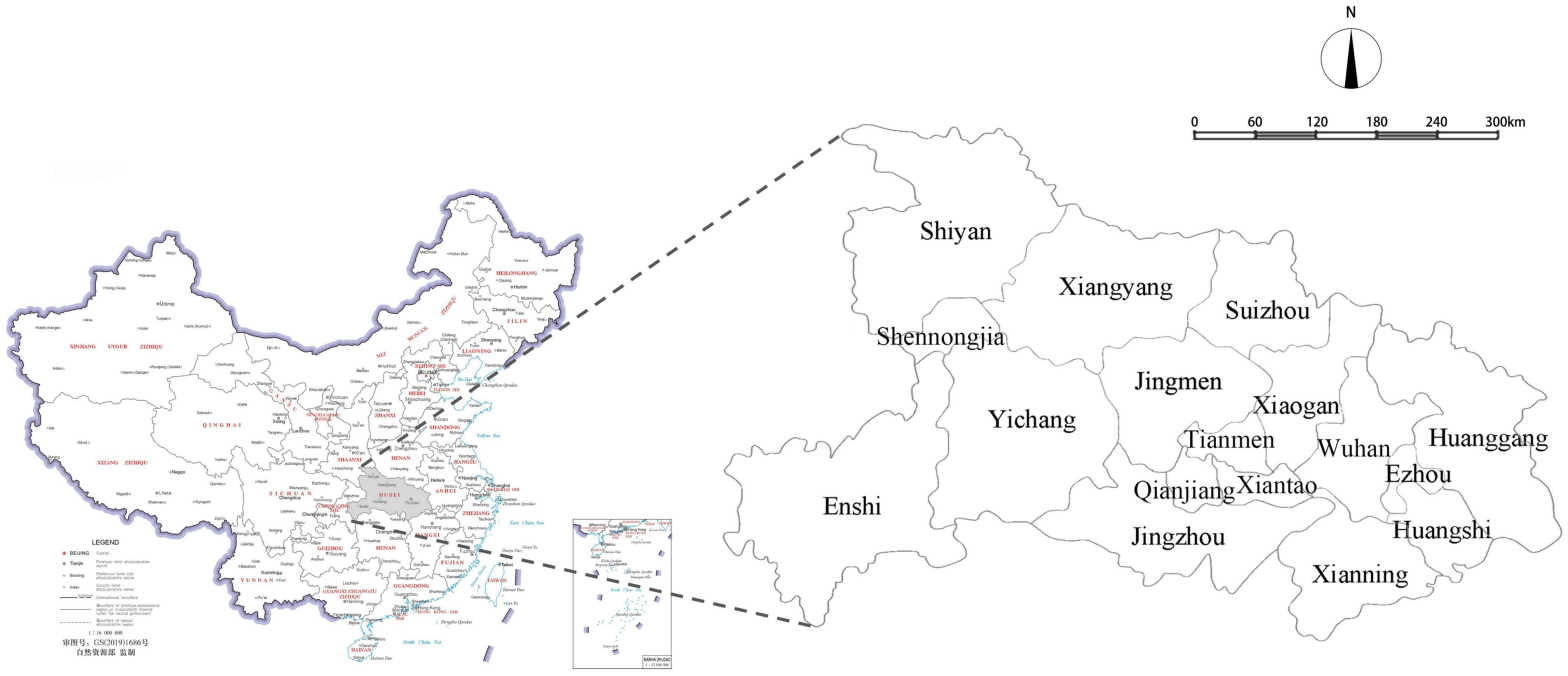
Locations of the Hubei province.

COVID-19 was first noted when a series of patients presented with pneumonia of unknown etiology from December 2019 to January 2020 in Wuhan, the capital city of Hubei Province (52), which has jurisdiction over 13 prefecture-level administrative regions (Wuhan, Huangshi, Shiyan, Yichang, Xiangyang, Ezhou, Jingmen, Xiaogan, Jingzhou, Huanggang, Xianning, Suizhou, Enshi), and four county-level administrative region units. In the past 10 years before the pandemic, there has been an increasing trend toward the new urbanization of prefecture-level cities (53). Therefore, the land cover in the province has changed overall, causing a series of social and ecological problems, including environmental pollution and biodiversity reduction (54). Before the city was blocked on January 23 2020, many residents moved from Wuhan to other cities to avoid infection, and COVID-19 cases at the early stage of the outbreak were not tested and accurately counted due to limited medical resources. The four county-level administrative region units are very small in size compared with the prefecture-level administrative regions. Therefore, 12 major cities (prefecture-level administrative regions except for Wuhan) in Hubei Province were selected as the sample sites. Their location is shown in Figure 2.

These 12 cities were subjected to the same strict closure policy for preventing the spread of COVID-19, including uniform rationing of daily food and receiving additional medical equipment and medical personnel, when COVID-19 was first identified in Wuhan. This situation has provided a series of comparable data to explore the relationships between land cover variables and the growth rate of COVID-19.

### 2.2 The data of COVID-19 growth rate cases collection

We collected the peak period of COVID-19 cases from the data released by the Health Commission of Hubei Province from February 1 to March 4, 2020, because all 12 cities were blocked on January 26, 2020, and the symptoms of COVID-19 infection appeared after an incubation period of approximately 5.2 days (55). On the other hand, no new COVID-19 cases in the 12 cities after March 4 were reported. The growth rate of COVID-19 cases was calculated using Equation (1):


$$G=\frac{N_{d}-N_{d-1}}{N_{d-1}}\times 100\%$$ (1)

In time series analysis, the weekly log-difference (Δlog(N + *ε*)) is a commonly used data processing method for dealing with non-stationarity and heteroscedasticity in time series data. This method achieves data stationarity and variance stabilization by taking the logarithm of the raw data and then calculating the difference between adjacent time points.

Specifically, the weekly log-difference was calculated using Equations (2, 3):


$$\Delta \log(N+\epsilon )=\log(N_{d}+\epsilon )-\log(N_{d-1}+\epsilon )$$ (2)

G≈Δlog(N)×100 (3)

Where, G: growth rate; N_d_ = Number of infection at day “d”; N_d-1_ = Number of infection at day “d-1” (the day before “d”); ϵ is a very small positive number, typically used to avoid taking the logarithm of zero or negative values. This is particularly important when dealing with real-world data, as the logarithm function is undefined for zero or negative values.

### 2.3 The data of land cover

The land cover data in Hubei Province was obtained from the third national land survey and the annual national land change survey in 2020 (Ministry of Natural Resources of China) (56). The third national land survey comprehensively adopted satellite remote sensing images with a resolution better than 1 meter as the base map for the survey. Government involvement, adequate financial support, and a rigorous validation system ensure the most systematic, accurate, comparable, and consistent land use data, which have recently been used by several academics (57, 58). And, we have also adopted the National Standard of the People’s Republic of China “Land Use Status Classification” (GB/T 21010-2007). To be more relevant to the topic of the study and facilitate discussion, we have adopted a first-level classification. All datasets used in this study were obtained from official provincial and municipal statistical and environmental reports to ensure accuracy and reliability (Table 1).

**Table 1:** Descriptive statistics for land cover of the 12 cities in Hubei Province.

Land cover variables	Min	Max	Mean	SD	Unit or formula	VIF Test	Tolerance	VIF thresholds
Wetland	283.52	14802.99	3650.076	3947.006	hectares (ha)	Retained	0.038	26.414
Cultivated Land	45758.93	689492.75	342335.138	201548.332	hectares (ha)	Retained	0.046	21.591
Orchard land	954.32	148030.52	39225.068	38945.709	hectares (ha)	Retained	0.382	2.618
Forest land	23375.37	1938412.18	734167.607	628768.534	hectares (ha)	Retained	0.027	37.294
Grassland	698.55	16089.24	6791.689	4402.875	hectares (ha)	Retained	0.059	16.917
Built-up land	26338.09	164124.85	95942.525	40102.776	hectares (ha)	Removed	NA	NA
Transportation land	6889.8	44831.15	24083.113	10701.818	hectares (ha)	Removed	NA	NA
Water body	34686.22	398449.57	130974.763	92398.168	hectares (ha)	Removed	NA	NA

Min, minimum; Max, maximum; SD, standard deviation.

### 2.4 The data of ancillary predictor variables

In recognition that various sociodemographic and environmental factors may confound the relationship between land cover and COVID-19 growth rates, we incorporated population density, age structure, socioeconomic indicators, healthcare capacity, and air pollution into the LASSO regression. Including these well-established determinants enables LASSO to penalize weak predictors and retain the most influential variables, thereby isolating the net effect of land cover with greater robustness. Population density data, including total population and land area, as well as socioeconomic indicators and healthcare service information, were collected from the Hubei Statistical Yearbook 2021 (Hubei Provincial Bureau of Statistics) (59). Demographic structure data were derived from the Bulletin of the Seventh National Population Census of Hubei Province (Hubei Provincial Bureau of Statistics) (60). Air pollution data were extracted from the Environmental Air Quality Report of Key Cities in Hubei Province (February 2020), published by the Hubei Provincial Department of Ecology and Environment (61). These sources provided comprehensive and standardized datasets that supported the comparative and spatial analyses conducted in this study.

### 2.5 Statistical analysis

Descriptive statistics were performed using IBM SPSS Statistics 27 to summarize the distributional characteristics of all variables. The variance inflation factor (VIF) was then computed to examine potential multicollinearity among the independent variables.

Furthermore, to discern the most salient determinants influencing the spatial variation of COVID-19 growth rates across 12 cities in Hubei Province, a Least Absolute Shrinkage and Selection Operator (LASSO) regression in RStudio (R version 4.5.1) was employed. Prior to modeling, all explanatory variables were standardized (mean = 0, standard deviation = 1) to ensure comparability of variable scales and to avoid biased penalization in the LASSO procedure. A Gaussian LASSO model (*α* = 1) was fitted using the glmnet package in R (62), and the optimal penalty parameter (*λ*.min) was determined by 10-fold cross-validation. Variables with non-zero coefficients at λ.min were considered significant contributors. Subsequently, a multiple linear regression was conducted using the selected variables to estimate standardized *β* coefficients, Wald χ^2^, *p*-values, and odds ratios (OR, 95% CI). This approach enables selective coefficient shrinkage, effectively excluding variables with weak explanatory power and mitigating multicollinearity among highly correlated land-use indicators.

Spatial autocorrelation was assessed using RStudio (R version 4.5.1) by calculating Moran’s I, a widely recognized statistic for quantifying spatial dependence among neighboring spatial units. This measure was employed to determine whether the spatial distribution of the study variables exhibited significant clustering, dispersion, or randomness. Furthermore, spatial econometric analyses were performed in RStudio using the spdep and spatialreg packages to account for spatial dependence in regression models. Specifically, the Spatial Autoregressive (SAR) model and the Spatial Lag of X (SLX) model were implemented to evaluate the influence of explanatory variables on the outcome variable while explicitly modeling spatial interactions across spatial units.

## 3 Results

The results of this study are presented in three main parts. First, the COVID-19 growth rate during the lockdown period was quantified across 12 cities in Hubei Province. Second, LASSO regression identified wetland, cultivated land, orchard land, forest land, and population density as the most influential predictors of the COVID-19 growth rate. Among these, wetland exhibited a significant positive association with the growth rate, whereas cultivated land showed a negative but marginally significant relationship. Orchard land and forest land both demonstrated weak negative effects. Finally, spatial dependence among the 12 cities was assessed using Moran’s I, and further analyses employing the Spatial Autoregressive (SAR) and Spatial Lag of X (SLX) models confirmed the robustness of the predictors identified through LASSO regression.

### 3.1 Overall evaluation of the growth rate of COVID-19

**Figure 3:**
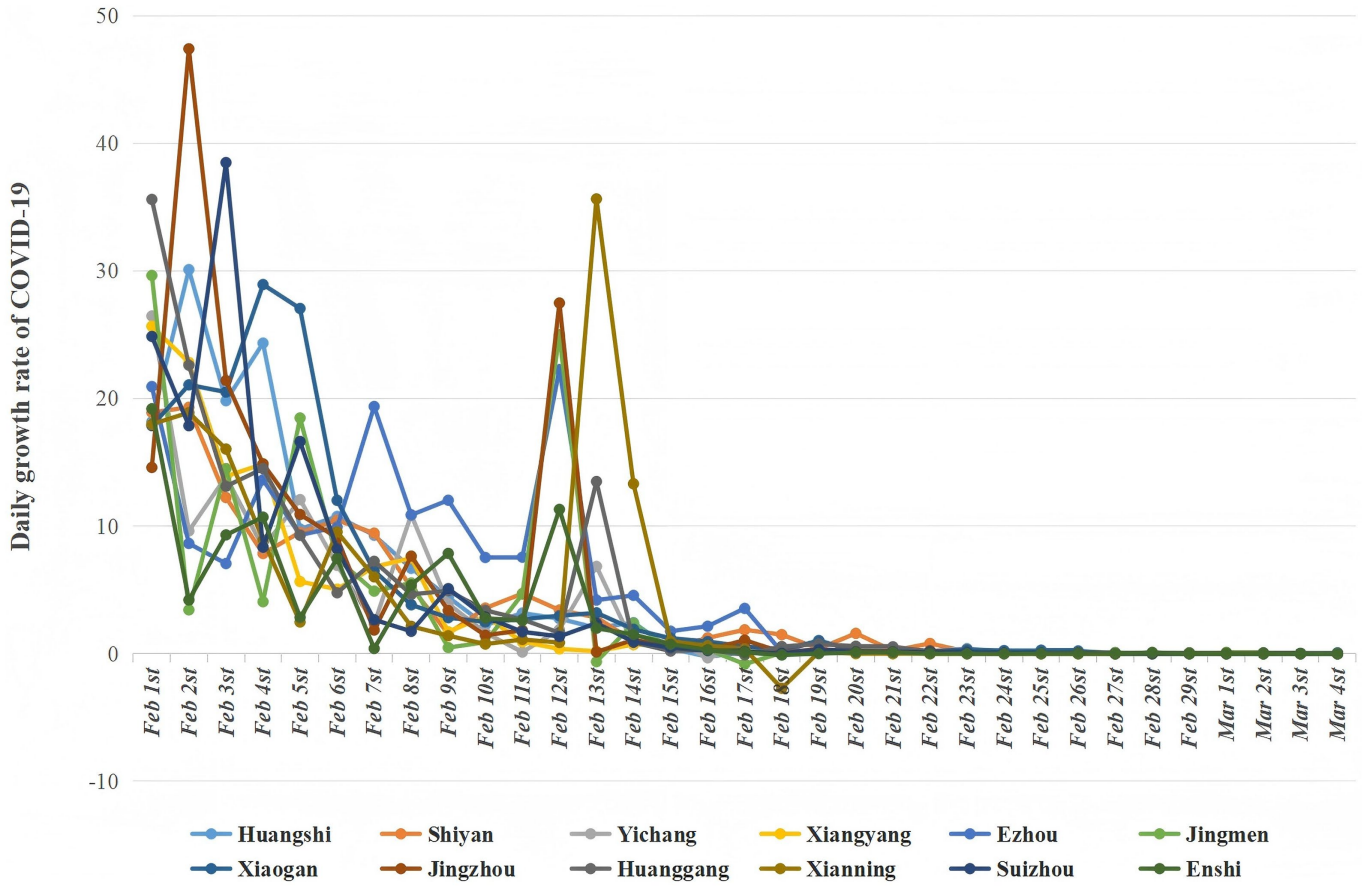
Daily growth rate of COVID-19 cases in 12 cities.

As of March 4, 2020, the cumulative number of reported COVID-19 cases in the 12 cities was as follows (n): Huanggang (*n* = 2,907), Xiaogan (*n* = 3,518), Suizhou (*n* = 1,307), Xiangyang (*n* = 1,175), Jingzhou (*n* = 1,580), Yichang (*n* = 931), Huangshi (*n* = 1,015), Jingmen (*n* = 928), Xianning (*n* = 836), Ezhou (*n* = 1,394), Shiyan (*n* = 672), and Enshi (*n* = 252). The mean growth rates of COVID-19 cases in the 12 sample cities are presented in Figure 3. Based on the average score, the highest growth rate was found in Ezhou (average score = 5.075), followed by Jingzhou (average score = 5.041) and Xiaogan (average score = 4.833). Enshi had the lowest growth rate (average score = 2.693).

### 3.2 LASSO-based variable selection

**Figure 4:**
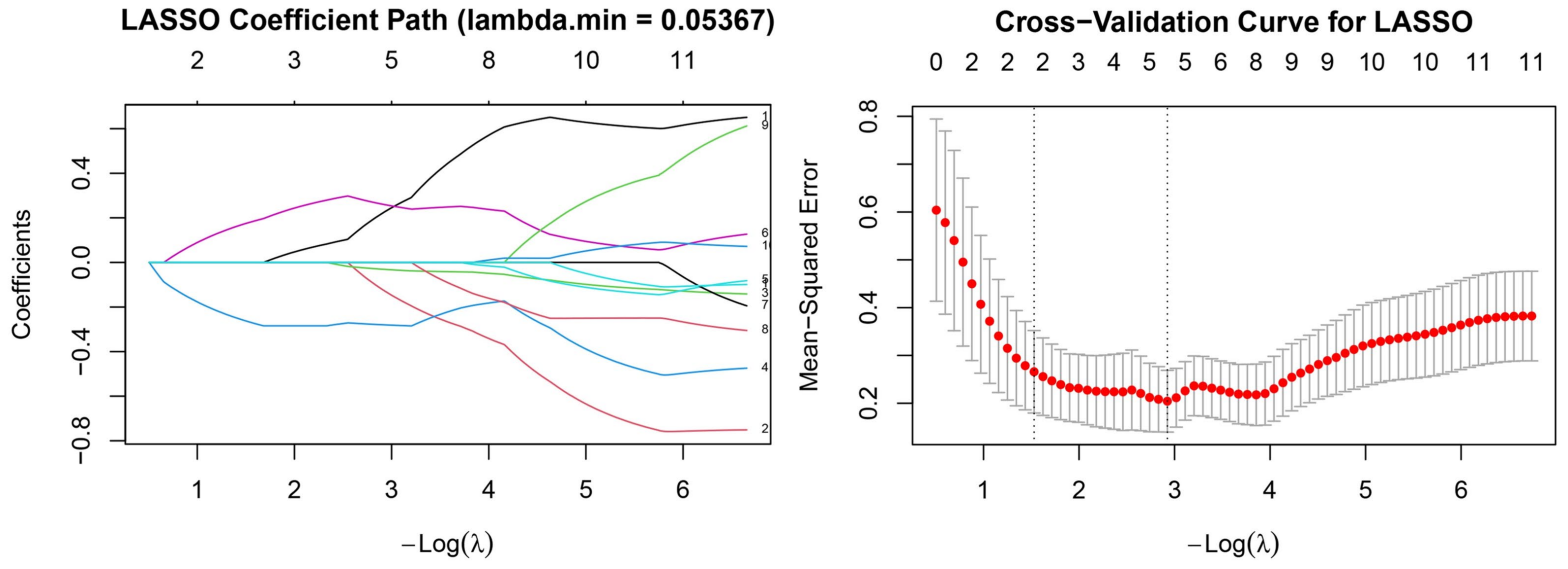
Lasso regression model screening variables.

To identify the most influential predictors associated with the regional of COVID-19 growth rates, the Least Absolute Shrinkage and Selection Operator (LASSO) regression was performed using standardized independent variables. As presented in Figure 4, the coefficient paths of all predictors gradually converged toward zero as the penalty parameter (*λ*) increased, reflecting the systematic elimination of variables with weak explanatory contributions. Variables with stable and nonzero trajectories near the optimal regularization level were considered to have a stronger association with the response variable. The 10-fold cross-validation curve (Figure 4) demonstrated that the mean-squared error (MSE) initially decreased with decreasing *λ*, reached its minimum at *λ* = 0.05367, and then increased again as overfitting began to occur. This λ value (λ min) was therefore selected as the optimal penalization threshold. At λ min = 0.05367, the LASSO model retained five predictors with nonzero coefficients: Wetland, Cultivated Land, Orchard Land, Forest Land, and Population Density. These retained variables were interpreted as the most relevant environmental and demographic factors influencing the spatial heterogeneity of COVID-19 growth rates among the 12 study cities. Their coefficient magnitudes indicate both the direction and strength of their effects, providing an interpretable foundation for subsequent multivariate regression analysis.

### 3.3 Multivariate regression analysis of selected predictors

A multivariate linear regression model was conducted using the predictors retained through the LASSO-based variable selection, including Wetland, Cultivated Land, Orchard Land, Forest Land, and Population Density (Table 2). The results demonstrated that Wetland coverage showed a statistically significant positive association with the COVID-19 growth rate (*β* = 0.494, *p* = 0.021). This finding indicates that cities with a higher proportion of wetlands tended to experience faster increases in infection rates. In contrast, Cultivated Land exhibited a negative association with the COVID-19 growth rate (*β* = −0.406, *p* = 0.051), showing a marginal level of significance. Although this relationship did not meet the conventional threshold of statistical significance, the direction of effect suggests that a greater proportion of cultivated land may mitigate infection spread, possibly due to lower population density or reduced human contact in such areas. The remaining variables—Orchard Land (*β* = −0.060, *p* = 0.287*), Forest Land (*β* = −0.300, *p* = 0.162*), and Population Density (*β* = 0.176, *p* = 0.210*)—did not exhibit statistically significant relationships with the COVID-19 growth rate. Nevertheless, their coefficient directions were consistent with theoretical expectations: Orchard Land and Forest Land both showed weak negative effects, implying potential ecological buffering roles, whereas Population Density displayed a positive trend, aligning with established evidence that higher population concentrations facilitate viral transmission (Table 3).

**Table 2:** Ancillary predictor variables of the 12 cities in Hubei Province.

Variable categories	Variables	Min	Max	Mean	SD	Unit or formula	VIF test	Tolerance	VIF thresholds
Population density	Population density	135.595	676.316	318.928	162.191	persons/km^2^	Retained	0.035	28.381
Age structure	Population aged 0–14 years	194,284	1,132,423	606,720	247,639	Population (n)	Retained	0.012	81.027
	Population aged 15–59 years	684,883	3,491,753	2,165,775	839,611	Population (n)	Removed	NA	NA
	Population aged 60 and above	200,188	1,256,495	742,384	333,219	Population (n)	Removed	NA	NA
	Population aged 65 and above	146,933	918,285	536,257	238,732	Population (n)	Removed	NA	NA
Socioeconomic indicators	Real estate investment	58.168	283.769	167.613	75.777	Billion yuan (CNY billion)	Removed	NA	NA
	Number of college students enrolled	0.782	9.830	4.643	2.382	10 thousand persons (10^4^ persons)	Retained	0.054	18.382
Healthcare capacity	Number of Healthcare Institutions	476	4,248	2358.417	1043.895	units	Removed	NA	NA
	Number of hospital beds	0.5847	3.9532	2.495	1.018	10 thousand beds (10^4^ beds)	Retained	0.015	65.576
Air pollution	Mean PM_10_ concentration (February)	37	61	53.333	6.060	Micrograms per cubic meter (μg/m^3^)	Retained	0.096	10.395
	Mean NO_2_ concentration (February)	8	21	13.25	4.146	Micrograms per cubic meter (μg/m^3^)	Retained	0.074	13.471

Min, minimum; Max, maximum; SD, standard deviation.

**Table 3:** Multivariate logistic regression analysis results of LASSO-selected variables.

Variable	Standardized β	Standard error	Wald *χ*^2^	OR (95% CI)	*p*-value
(Intercept)	4.044267677	0.084439714	2293.962314	57.069 (48.364–67.341)	5.5535E-09
Wetland	0.49387989	0.160010538	9.526766333	1.639 (1.198–2.242)	0.02148246
Cultivated land	−0.405517723	0.16714064	5.886478426	0.667 (0.480–0.925)	0.051427056
Orchard land	−0.059882552	0.116368013	0.264809378	0.942 (0.750–1.183)	0.625234407
Forest land	−0.299988887	0.17633954	2.894082633	0.741 (0.524–1.047)	0.139807777
Population density	0.175095285	0.164371084	1.13474444	1.191 (0.863–1.644)	0.327743725

### 3.4 Analysis of spatial autocorrelation

To assess the presence of spatial autocorrelation in the model residuals, we performed Moran’s I test under randomization using the spatial weights matrix. The results indicated no significant spatial autocorrelation, with a Moran’s I statistic of −0.0893 (Expectation = −0.0909, Variance = 0.0286; standardized deviate = 0.0093, *p* = 0.4963). These findings confirm the absence of significant spatial dependence in the residuals, confirming that the model effectively accounts for spatial structure and supporting the robustness of the regression estimates.

The Spatial Autoregressive (SAR) model revealed no significant spatial dependence across the 12 cities in Hubei Province. The estimated spatial autoregressive coefficient was *ρ* = −0.107 (*p* = 0.518), and the Lagrange Multiplier test for residual autocorrelation was also non-significant (*p* = 0.646). These results indicated that spatial autocorrelation was weak and that adding a spatial lag term did not improve model performance. Among the explanatory variables, only wetland (*β* = 0.476, *p* < 0.001), cultivated land (*β* = −0.416, *p* < 0.001), and forest land (*β* = −0.321, *p* = 0.011) remained statistically significant, consistent with the variables selected by the LASSO regression.

Similarly, the Spatial Lag of X (SLX) model did not detect any significant direct or indirect (lagged) effects of neighboring land-use factors (all *p* > 0.05). The absence of significant spatial spillover effects further supports the conclusion that the relationships identified in the non-spatial model were not driven by spatial dependence.

Overall, both the SAR and SLX models consistently demonstrated that the spatial dependence of COVID-19 growth rates was negligible. These findings confirmed the robustness of the LASSO-based variable selection results, and indicated that the associations between land-use factors and COVID-19 growth were primarily local and not influenced by spatial autocorrelation (Table 4).

**Table 4:** Comparative results of SAR and SLX spatial models.

Variable	SAR estimate	SAR Std. error	SAR *p*-value	SLX Estimate	SLX Std. error	SLX *p*-value
Intercept	4.4741	0.6649	<0.001 ***	3.8748	0.1453	0.024 *
Wetland	0.4762	0.1123	<0.001 ***	0.1155	0.344	0.794 n.s.
Cultivated Land	−0.4158	0.1184	0.00044 ***	0.5645	0.4674	0.440 n.s.
Orchard Land	−0.0506	0.0824	0.539 n.s.	−0.3921	0.2095	0.312 n.s.
Forest Land	−0.3209	0.1256	0.011 *	0.8507	0.5523	0.367 n.s.
Population Density	0.1907	0.1166	0.102 n.s.	1.3679	0.6328	0.276 n.s.
Spatial Lag (ρ)	−0.107	0.1656	0.518 n.s.	–	–	–
lag(Wetland)	–	–	–	−2.261	1.1021	0.289 n.s.
lag(Cultivated Land)	–	–	–	3.3385	1.3171	0.239 n.s.
lag(Orchard Land)	–	–	–	−0.8541	0.7509	0.459 n.s.
lag(Forest Land)	–	–	–	2.6338	1.7421	0.372 n.s.
lag(Population Density)	–	–	–	3.4562	1.9758	0.331 n.s.
Model Fit (AIC)	11.849	–	–	–	–	–
Spatial Autocorr. (LM)	0.211	–	0.646 n.s.	–	–	–
Observations	12	–	–	12	–	–

*p < 0.05, ***p < 0.001. n.s., not significant. SAR, Spatial Autoregressive Lag Model; SLX, Spatial Lag of X Model.

## 4 Discussion

The emergence of COVID-19 has highlighted the growing need to reconsider land cover in support of sustainable habitat development. Within the current OH perspective, it is crucial not only to elucidate the potential impacts of land cover but also to investigate the interactions among the environment, humans, animals, and plants, to mitigate disease transmission in future scenarios.

Among all environmental variables, wetland coverage exhibited a significant positive association with COVID-19 growth rate. This result differs from most studies in environmental health, where urban blue spaces and proximity to water are generally associated with health benefits (63), and visiting wetland parks are reported to confer greater mental than physical health benefits (64). Previous literature concludes that wetlands can pose risks to human health through disease transmission and the release of pollutants (65). The observed positive association between wetlands and epidemic growth in this study likely reflects the combined effects of hydrological conditions, water pollution, and human activities. Untreated wastewater represents a potential route for SARS-CoV-2 transmission to recreational waters (66). Previous studies indicate that SARS-CoV can persist in untreated wastewater at 4 °C for up to 14 days, despite the fact that survival decreases to 2 days at 20 °C (67). Similar concerns have been raised for SARS-CoV-2, with studies from the Netherlands (68), Australia (69), India, Italy (70), and France (71) detecting SARS-CoV-2 RNA in untreated sewage. SARS-CoV-2 RNA has been detected in multiple sewage systems and environmental samples in Wuhan (72). Risk assessments along the Yangtze, Han, and Fu River basins in Hubei further indicate that riverine cities exhibit varying COVID-19 transmission risks, with differences in safety radius and timing (73). Rivers may act as transmission vectors, carrying viruses downstream and posing health risks to basin cities (74). Hydrological events such as rainfall and snowmelt can exacerbate combined sewer overflows, leading to substantial discharges of untreated sewage into natural water bodies (75), which in turn increases the flux of infectious RNA bacteriophages, posing a potential threat to surface water (76). Exposure to urban floodwater has been quantitatively linked to infection risks from waterborne pathogens, with up to 33% of children at risk and some risk for adults (77). Carducci et al. (78) emphasized the urgent need to understand coronavirus presence, persistence, and public health implications in aquatic environments. Taken together, these findings indicate that wetlands, while providing important ecological and recreational benefits, can also facilitate the persistence and transmission of viruses, particularly when impacted by pollution or urban wastewater. The hydrological and microbial characteristics of these environments, in combination with human use, may contribute to the spread of COVID-19. These observations highlight the critical importance of systematic wetland monitoring and management as part of strategies to mitigate potential public health risks during epidemic outbreaks.

This study, initiatively, identified a negative relationship between agricultural land use and the growth rate of COVID-19. Among the variables selected by the LASSO regression, both cultivated land and orchard land exhibited negative associations, although neither reached statistical significance. Nevertheless, the consistent direction of these effects suggests a potential suppressive influence of agricultural landscapes on epidemic acceleration. Cultivated and orchard areas are generally characterized by open spatial structures, low population density, and good air circulation, factors that collectively reduce close interpersonal contact and limit the accumulation of virus-laden aerosols. These regions also tend to have lower population mobility and weaker transportation connectivity than urban centers, which may further slow the spatial diffusion of infectious diseases. In addition, agricultural landscapes are associated with seasonal and localized labor activities (79), which constrain the frequency and duration of human gatherings and may weaken potential transmission chains. These findings indicate that the negative associations between cultivated and orchard lands and the COVID-19 growth rate may reflect the passive ecological effects of agricultural landscapes, whereby low population density, limited mobility, and favorable natural ventilation jointly mitigate the spatial spread of infections.

Forest land in the present analysis exhibited a weak negative effects with the growth rate of COVID-19. This finding aligns with the findings of numerous studies, suggesting that, in the context of COVID-19, exposure to forests and natural environments can alleviate pandemic-related psychological stress, highlighting the importance of “green prescriptions” that encourage engagement with vegetated areas (80–82). Beyond these mental health benefits, the consistent negative direction of the association supports the hypothesis that natural vegetation may provide ecological buffering against epidemic spread, in line with previous research which concludes that COVID-19 severity is influenced not only by population density but also by environmental factors such as air quality and vegetation cover (83). Forested areas typically offer stable microclimates, cleaner air, and lower population density, which may reduce viral survival and transmission risk. Research suggests that declining forests increase the likelihood of pathogen transmission across species barriers (84). Thus, a reduction in forests may increase the transmission of diseases among different species, increasing the incidence of zoonotic diseases such as COVID-19. Moreover, Mediterranean forest species release biogenic volatile organic compounds (VOCs) that can enhance immune function and exhibit antiviral activity. For example, *Laurus nobilis* L. essential oil, rich in *α*-pinene, β-pinene, β-ocimene, and 1,8-cineole, has demonstrated antiviral effects against SARS-CoV-1, suggesting potential relevance for SARS-CoV-2 (85). Overall, although the effect of forest coverage on COVID-19 growth rate was minor and non-significant, the persistent negative trend between the two factors, together with ecological, biochemical, and public health evidence, underscores the potential of forests to enhance regional epidemic resilience. Preservation and expansion of forested areas, particularly evergreen and VOC-rich species, may thus support both ecosystem health and community well-being (83, 86).

Population density, identified by LASSO regression as a key variable, showed a positive but non-significant association with COVID-19 growth rate. Although not statistically significant, the positive direction is consistent with epidemiological theory and prior studies, indicating that dense populations may facilitate viral transmission through higher contact rates, social interactions, and mobility (44, 87–89). Multiple studies report that overcrowded areas with concentrated commercial and transportation networks are more susceptible to infection, even when infrastructure and healthcare are well developed (33, 90). Accordingly, the influence of density should be interpreted within a broader urban and environmental context rather than as an isolated driver. Despite its non-significance, the observed positive association supports the role of population density as a potential risk factor for epidemic spread.

## 5 Recommendations

The OH provides us with a comprehensive perspective to rethink the health benefits of sustainable human habitat, and implicate for future studies should emphasize not only the symbiotic relationship between humans and nature, but also the need for humans to actively participate in interactions among humans, animals, and the environment to promote overall sustainable habitat development.

First of all, this study provides empirical evidence to enhance the understanding of the spread of COVID-19 from an environmental perspective, offering valuable insights for policy development. Since the 19th century, there have been four outbreaks of cholera in the United Kingdom, and it was not until the enactment of the Public Health Act that the situation with infectious diseases gradually improved (91). This historical lesson underscores the pivotal role of environmental governance policies in safeguarding public health. From this perspective, two key implications emerge. First, it is proposed to formulate corresponding policies from an environmental perspective. Environmental health constitutes a fundamental basis for the health and well-being of humans, animals, and plants. Protecting natural environments and maintaining ecosystem integrity can help preserve biodiversity, limit the emergence and transmission of diseases at the human–animal–plants interface (e.g., via the dilution effect), enhance well-being, and promote overall health. Second, effective policy requires active human engagement with the environment, highlighting the need to enact regulations that protect natural and ecological systems. Specifically, policies should prioritize the enhancement and conservation of blue–green infrastructure. As demonstrated by this study, natural elements such as forests and wetlands can serve as ecological buffers against the transmission of infectious diseases. The recommendations are explicated from planning and design levels as following:

At the planning level, this study indicates that natural and agricultural landscapes—including forests, wetlands, cultivated land, and orchards—may exert a modest ecological buffering effect against the spread of infectious diseases. Future spatial planning should prioritize sustainable land cover management within the OH perspective, integrating environmental health considerations into land use decisions. To solve the problems brought about by the wave of urbanization and industrialization, planning methods such as garden cities (92) and satellite cities (93) have been proposed to provide a beneficial and healthy habitat for people. Similarly, urban planners are encouraged to optimize spatial structures by incorporating green infrastructure—particularly wetlands, forests, and agricultural landscapes. Additionally, forests should be prioritized for protection, and wetlands should be systematically monitored. Policymakers need to implement preventive measures, including the surveillance and control of water pollution in environments that may facilitate the spread of infectious diseases such as COVID-19 (94), while forest conservation helps preserve ecological integrity and health benefits. Urban planners should also adopt strategies to regulate population density and manage human mobility through deliberate interventions. Collectively, these strategies can foster a sustainable, resilient, and health-oriented spatial structure that aligns environmental protection with epidemic prevention objectives.

At the design level, several strategies can enhance the resilience of human habitats to infectious disease risks. First, given that high population density substantially increases the likelihood of viral transmission, public space design should prioritize ventilation and greening to mitigate this effect. Open spatial layouts, abundant vegetation, and improved air circulation can help dilute airborne particles and reduce close-contact risks in densely populated environments. Second, integrating agricultural landscapes into residential areas can strengthen community resilience by providing outdoor spaces that disperse human gatherings, promote physical and mental well-being, and serve as potential supplementary food sources during epidemic disruptions. Third, multifunctional blue–green spaces should be incorporated at community, park, and neighborhood scales to combine functions of recreation, hygiene, psychological restoration, physical activity, and ecological services, thus achieving synergistic benefits for both public health and the environment (95–97). Overall, beyond emphasizing human–nature coexistence, it is essential to encourage active human participation in environmental design and management, fostering sustainable, health-oriented habitats through interactions among people, animals, and plants.

## 6 Limitations

This study has several limitations and should be further studied in the future. First, the analysis was conducted based on only 12 sample cities within Hubei Province, which may limit the generalizability of the findings. The relatively small number of samples could also increase the risk of multicollinearity among land cover variables, potentially influencing the model’s explanatory power. Future studies should expand the spatial scope and include more diverse regions, thereby enhancing the robustness and representativeness of the results. Second, the temporal scope represents another limitation. We analyzed the COVID-19 growth rate only during the most severe month of the outbreak, without considering a longer observation period. This limited time frame may fail to capture dynamic changes in epidemic patterns or the potential lagged effects of environmental exposure. Future studies should incorporate long-term time-series data and multiple epidemic phases to better identify sustained or delayed associations between land cover, population factors, and the evolution of epidemic transmission. Third, there was a lack of demographic information on confirmed COVID-19 cases in the 12 cities. Government published data did not include individual-level characteristics such as gender, age, or chronic disease status. Potential reporting errors during the early outbreak, coupled with the absence of demographic, clinical, mobility, and population-related covariates, may have influenced the robustness of the results. Future research should consider regions with more comprehensive demographic and health datasets to better elucidate the interactions between land cover patterns, population characteristics, and COVID-19 transmission dynamics. Fourth, this study adopted a first-level classification of land cover, which is relatively coarse and represents an exploratory stage in this research direction. Future work could utilize a finer-grained classification framework to examine specific land cover subtypes, providing more detailed insights into how ecological heterogeneity influences epidemic dynamics. For instance, the relationships between different forest subtypes and emerging infectious diseases remain largely unexplored (98). Such approaches could also offer more precise guidance for urban planners and policymakers aiming to integrate environmental and public health considerations into spatial decision-making. Finally, this study is subject to the ecological fallacy, as the analysis was conducted at the city level. Consequently, associations observed between land cover characteristics and COVID-19 growth rates cannot be directly interpreted as causal relationships at the individual level.

## 7 Conclusion

This study aimed to elucidate the effects of land cover on COVID-19 growth rates and, positions the environment as a systematically underappreciated and essential aspect of sustainable habitats from the OH perspective, which coordinates interactions among humans, animals, and plants. The results indicate that wetland exhibited a significant positive association with COVID-19 growth rates, whereas cultivated land showed a marginally significant negative relationship, and orchard and forest lands displayed weak negative effects. These results underscore the preventive role of the environment, supporting the recommendation that policymakers, planners, and designers adopt a holistic, integrated, and unified approach that balances human, animal, and environmental health in addressing complex health and ecological challenges.

Based on our findings, this study introduces an environmentally grounded pathway to proactive health and a non-medical preventive mechanism for infectious-disease mitigation, thereby extending the theoretical scope of the One Health and providing a stronger basis for sustainable habitat management. One Health continues to evolve, it becomes essential not only to reveal the potential influences of land cover on disease dynamics but also to identify environmental mechanisms capable of preventing or mitigating infectious-disease transmission in an increasingly uncertain future. Our findings align with the broad scientific consensus that ongoing human activities are irreversibly degrading ecosystem integrity, thereby creating conditions that facilitate the emergence of novel infectious diseases (99). Given that human interventions shape disease risks both directly and indirectly, these insights underscore the urgent need to integrate sustainable land-cover policies, spatial planning strategies, and habitat-design approaches that explicitly consider infection sources, transmission pathways, and susceptible populations. Such efforts are indispensable for strengthening environmental preparedness and advancing sustainable public-health protection under the One Health perception.

Today, 5 years after the outbreak, many of these environment gains in short-term emissions reduction and energy use caused by the epidemic have diminished, and these environment changes have prompted rethinking (97). The most basic way to prevent the spread of disease is to restore the balance between human beings and nature in the human habitat to create a sustainable habitat. Its essential to recognize that we are in the era of “One World, One Health.” In the processes of environment change we recommend the promotion of public health policies adapted to the development of the environment, the establishment of sustainable planning, and the designs that support a healthy environment to enhance well-being.

## Data Availability

The original contributions presented in the study are included in the article/Supplementary material, further inquiries can be directed to the corresponding author.
