# Supplementary material for: Does land cover affect the growth rate of COVID-19? Rethinking sustainable habitat from the One Health perspective using data from 12 cities during lockdown in Hubei Province, China
Source: Front Public Health. 2026 Jan 14;13:1682820. doi: 10.3389/fpubh.2025.1682820 (PMC12848805; doi:10.3389/fpubh.2025.1682820)
Supplement: Supplementary file 1 [file Table_1.docx]

# =========================================================

# Complete Workflow: Standardized LASSO Regression + Coefficient Path Plot

# =========================================================

# Install packages (comment out if already installed)

# install.packages(c("openxlsx","glmnet","dplyr","broom","readxl"))

library(openxlsx)

library(glmnet)

library(dplyr)

library(broom)

library(readxl)

# ------------------------------

# 1️⃣ Load data

# ------------------------------

data <- read.xlsx("data.xlsx") # Assume the first column is growthrate

y <- as.double(data$growthrate)

X <- data[, -1]

# ------------------------------

# 2️⃣ Standardize predictors

# ------------------------------

X_scaled <- scale(X)

X_matrix <- as.matrix(X_scaled)

# ------------------------------

# 3️⃣ Fit LASSO regression (Gaussian model)

# ------------------------------

set.seed(123)

fit <- glmnet(X_matrix, y, family = "gaussian", nlambda = 1000, alpha = 1)

# ------------------------------

# 4️⃣ Basic plot: coefficient path

# ------------------------------

plot(fit, xvar = "lambda", label = TRUE)

title("LASSO Coefficient Path (Gaussian Model)", line = 2.5)

# ------------------------------

# 5️⃣ Cross-validation to select optimal lambda

# ------------------------------

cvfit <- cv.glmnet(X_matrix, y, family = "gaussian", alpha = 1)

lambda_min <- cvfit$lambda.min

cat("✅ Optimal lambda.min =", lambda_min, "\n")

# Mark lambda.min on path plot

plot(fit, xvar = "lambda", label = TRUE)

abline(v = log(lambda_min), col = "red", lty = 2)

title(sprintf("LASSO Coefficient Path (lambda.min = %.4g)", lambda_min), line = 2.5)

# ------------------------------

# 6️⃣ Extract non-zero coefficients at lambda.min

# ------------------------------

coef_min <- coef(cvfit, s = "lambda.min")

coef_min_mat <- as.matrix(coef_min)

coef_vec <- as.vector(coef_min_mat)

var_names <- rownames(coef_min_mat)

nonzero_idx <- which(coef_vec != 0)

active_vars <- var_names[nonzero_idx]

active_vars <- active_vars[active_vars != "(Intercept)"]

cat("✅ Variables selected by LASSO:\n")

print(active_vars)

# ------------------------------

# 7️⃣ Compute effect sizes via linear regression (if variables selected)

# ------------------------------

if (length(active_vars) > 0) {

formula_str <- paste("growthrate ~", paste(active_vars, collapse = " + "))

df_model <- data.frame(growthrate = y, X_scaled)

model <- lm(as.formula(formula_str), data = df_model)

results <- tidy(model) %>%

mutate(

`Wald χ²` = (statistic)^2,

OR = exp(estimate),

OR_lower95 = exp(estimate - 1.96*std.error),

OR_upper95 = exp(estimate + 1.96*std.error),

`Standardized β` = estimate

) %>%

select(term, `Standardized β`, std.error, `Wald χ²`, OR, OR_lower95, OR_upper95, p.value) %>%

rename(

Variable = term,

`Standard Error` = std.error,

`P-value` = p.value

)

# SCI-style OR column

results <- results %>%

mutate(`OR (95% CI)` = sprintf("%.3f (%.3f–%.3f)", OR, OR_lower95, OR_upper95)) %>%

select(Variable, `Standardized β`, `Standard Error`, `Wald χ²`, `OR (95% CI)`, `P-value`)

print(results)

# Export Excel file

write.xlsx(results, "LASSO_SCI_table.xlsx", rowNames = FALSE)

cat("✅ Table generated: LASSO_SCI_table.xlsx\n")

} else {

cat("⚠️ No variables selected at lambda.min. Skipping linear regression and effect size calculation.\n")

}

# ------------------------------

# 8️⃣ Plot cross-validation curve (CV curve for LASSO)

# ------------------------------

plot(cvfit)

title("Cross-Validation Curve for LASSO", line = 2.5)

abline(v = log(lambda_min), col = "red", lty = 2)

text(log(lambda_min), min(cvfit$cvm), labels = sprintf("lambda.min=%.4g", lambda_min),

pos = 3, col = "red")

# ======================================

# Spatial Autocorrelation Test & Spatial Regression (SAR / SLX)

# ======================================

# Install necessary packages (comment out if already installed)

# install.packages(c("spdep", "spatialreg", "dplyr"))

library(spdep)

library(spatialreg)

library(dplyr)

# ------------------------------

# 1️⃣ Construct spatial weight matrix

# ------------------------------

# Assume adj_mat is a 12×12 adjacency matrix (1 = adjacent, 0 = not adjacent)

# Row-standardize the matrix

listw <- mat2listw(adj_mat, style = "W")

# ------------------------------

# 2️⃣ Build data frame

# ------------------------------

df_model <- data.frame(growthrate = y, X_scaled)

# Keep only LASSO-selected variables

if(length(active_vars) == 0){

stop("⚠️ No variables selected by LASSO; spatial regression cannot be performed.")

}

formula_str <- paste("growthrate ~", paste(active_vars, collapse = " + "))

formula_lm <- as.formula(formula_str)

# ------------------------------

# 3️⃣ Compute Moran’s I for residuals

# ------------------------------

lm_model <- lm(formula_lm, data = df_model)

residuals_lm <- residuals(lm_model)

moran_test <- moran.test(residuals_lm, listw)

cat("=== Moran's I test results ===\n")

print(moran_test)

# ------------------------------

# 4️⃣ Spatial regression: SAR model

# ------------------------------

sar_model <- lagsarlm(formula_lm, data = df_model, listw = listw)

cat("=== SAR spatial regression results ===\n")

summary(sar_model)

# ------------------------------

# 5️⃣ Spatial regression: SLX model (optional)

# ------------------------------

# SLX adds spatially lagged predictors W*X

slx_model <- lmSLX(formula_lm, data = df_model, listw = listw)

cat("=== SLX spatial regression results ===\n")

summary(slx_model)

# ------------------------------

# 6️⃣ Export results (optional)

# ------------------------------

# SAR model coefficients

sar_coef <- summary(sar_model)$Coef

write.xlsx(as.data.frame(sar_coef), "SAR_results.xlsx", rowNames = TRUE)

cat("✅ SAR regression results exported: SAR_results.xlsx\n")

# SLX model coefficients

slx_coef <- summary(slx_model)$Coef

write.xlsx(as.data.frame(slx_coef), "SLX_results.xlsx", rowNames = TRUE)

cat("✅ SLX regression results exported: SLX_results.xlsx\n")
